# Real microgravity condition promoted regeneration capacity of induced pluripotent stem cells during the TZ‐1 space mission

**DOI:** 10.1111/cpr.12574

**Published:** 2019-02-06

**Authors:** Jin Zhou, Xiao‐Hui Dong, Feng‐Zhi Zhang, Hui‐Min Zhu, Tong Hao, Xiao‐Xia Jiang, Wei‐Bo Zheng, Tao Zhang, Pei‐Zhe Wang, Hong Li, Jie Na, Chang‐Yong Wang

**Affiliations:** ^1^ Tissue Engineering Research Center, Institute of Military Cognition and Brain Sciences Academy of Military Medical Sciences Beijing China; ^2^ Shanghai Institute of Technical Physics Chinese Academy of Sciences Shanghai China; ^3^ School of Medicine Tsinghua University Beijing China

**Keywords:** bioreactor, induced pluripotent stem cells, regeneration, space microgravity

## Abstract

Induced pluripotent stem cells (iPSCs) are reprogrammed somatic cells that gained self‐renewal and differentiation capacity similar to embryonic stem cells. Taking the precious opportunity of the TianZhou‐1 spacecraft mission, we studied the effect of space microgravity (µg) on the self‐renewal capacity of iPSCs. Murine iPSCs carrying pluripotency reporter Oct4‐GFP were used. The Oct4‐EGFP‐iPSCs clones were loaded into the bioreactor and exposed to μg in outer space for 14 days. The control experiment was performed in identical device but on the ground in earth gravity (1 g). iPSCs clones were compact and highly expressed Oct4 before launch. In μg condition, cells in iPSC clones spread out more rapidly than those in ground 1 g condition during the first 3 days after launch. However, in 1 g condition, as the cell density increases, the Oct4‐GFP signal dropped significantly during the following 3 days. Interestingly, in μg condition, iPSCs originated from the spread‐out clones during the first 3 days appeared to cluster together and reform colonies that activated strong Oct4 expression. On the other hand, iPSC clones in 1 g condition were not able to recover Oct4 expression after overgrown. Our study for the first time performed real‐time imaging on the proliferation process of iPSCs in space and found that in μg condition, cell behaviour appeared to be more dynamic than on the ground.

Dear Editor,

The rapid development of space technology enabled long‐term human travel, living and potentially reproduction in the outer space; therefore, it is of practical significance to study the effects of microgravity (µg) on cells, organisms and even individuals. Induced pluripotent stem cells (iPSCs) can be generated from differentiated adult cells, they can be maintained at undifferentiated state indefinitely in vitro and can develop to all types of functional cells in the body, thus can be used as a platform for personalized drug test, and regeneration therapies.^1^ Both cell identities and the microenvironment have significant impact on their physiological functions. One direct effect of µg on cells was the changes of cytoskeleton and adhesion, which were involved in cell morphology and intercellular communications. Therefore, long‐term exposure to μg could have profound effect on the functions of all human cells in the body.^2^ Space µg has incomparable particularity to simulated µg in some degree. Taking the opportunity of TianZhou (TZ)‐1 spacecraft mission, we investigated the effect of space µg environment on the self‐renewal capacity of iPSCs.

The Space Bioreactor System in TZ‐1 cargo spacecraft was designed and constructed by Shanghai Institute of Technical Physics, Chinese Academy of Sciences (Figure S1). It was engineered to perform long‐term cultivation of mammalian cells, including the replacement of fresh medium to different microfluidic cell culture chambers at desired time interval, maintenance of constant temperature. More importantly, the bioreactor was also equipped with two microscopes and camera module to record both bright‐field and fluorescence images. The camera took images of different fields of cells in the cell culture units automatically or according to the uploaded control commands.

To monitor the self‐renewal status of iPSCs in space, we obtained embryonic fibroblast cells from Oct4‐GFP reporter mice and reprogrammed them to iPSCs (Figure S2).^3^ Oct4‐GFP‐iPSCs clones were loaded onto the bioreactor, and then integrated onto TZ‐1 spacecraft, which was launched on 20th April 2017. TZ‐1 took 603 seconds to reach its orbit at 380 kilometres above the earth, then flied for 5 months in space. The ground 1 g control experiment was performed at Tsinghua University under identical conditions except for exposure to 1 g on earth.

The iPSCs clones were plated near the centre of the cell culture units to ensure the exposure of sufficient nutrition from medium. For real‐time imaging in space, a 5× magnification objective was used to monitor whole field of the culture unit (Figure 1A, B, Figure S3A). The iPSCs clones from the μg and 1 g groups were similar in size at the starting point of −5.5 hours (Figure 1A, first column). During day (D) 1‐3, iPSCs in both groups grew bigger gradually, but the area covered by clones of μg group was significantly larger than that of 1 g group [Figure 1B, C]. Interestingly, under μg condition, cells from the iPSCs clone spread out much more extensively from D2.

**Figure 1:**
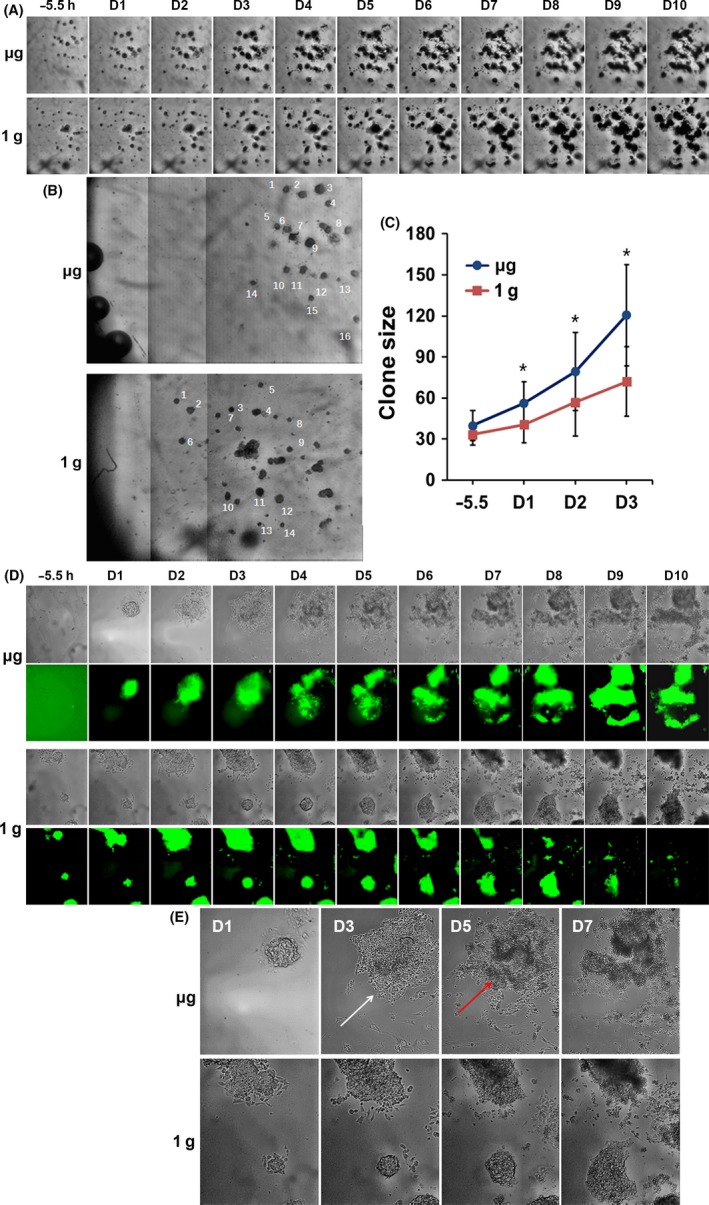
Microgravity promoted the regeneration capacity of iPSCs. A, Representative time‐lapse images of Oct4‐GFP iPSCs in on‐orbit μg and ground control 1 g condition during 10 days of experiment (5ˣ magnification). Bar = 400 μm. B, 3 images of different fields were taken every day and multiple iPSCs clones were selected. C, Line graph representation of the size of iPSCs clone in μg and 1 g until D3. * *P* < 0.05. D, Representative time‐lapse fluorescence images of Oct4‐GFP iPSCs in μg and 1 g during 10 days of experiment (20ˣ magnification). Bar = 100 μm. E, The enlarged images of iPSCs in μg and 1 g group. In the μg condition, some cells migrated out from the iPSCs clones (white arrow), cells migrated out from the original colony formed cluster (red arrow)

Another 20× magnification objective was used to monitor cell morphology and fluorescence (Figure 1D, E, Figure S3B). In the on‐orbit experiment, iPSCs clones were compact and highly expressed Oct4 at D1 after launch (Figure 1D). Then, some cells migrated out from the iPSCs clones at D2 and D3 (Figure 1E, white arrow). The iPSCs clones downregulated the expression of pluripotent marker Oct4 after D4, and that was the reason we only compared the regeneration capacity of iPSCs during D1‐D3 (Figure 1C). Interestingly, cells migrated out from the original colony formed cluster on D4, and from that day onwards (Figure 1E, red arrow), the Oct4‐GFP signal increased as the cell cluster grew bigger. While in the ground control 1 g experiment, few cells spread out from the colony. The Oct4‐GFP fluorescence suddenly dropped from D6 after launch and was not able to recover after overgrown (Figure 1D). Above data suggested that the μg condition leads to more dynamic behaviour of iPSCs, while they can still maintain pluripotency.

The opportunities of spacecraft mission were very limited. Between 1980 and 1996, vertebrate animals were aboard the Soviet (Russian) biosatellites seven times to study the influence on hematopoietic tissues. They found that the number of stromal fibroblastic progenitor cells and hematopoietic progenitor cells in the rat bone marrow were significantly decreased when animals were exposed to spaceflight conditions.^4^ Very few experiments at cellular level had been done due to the difficulties in performing cell culture in space. Previous study carried out by a NASA mission found that exposure to µg preserves greater stemness of mouse embryonic stem cells and inhibited their differentiation.^5^ However, there were no images of cells from their study; thus, it was hard to know how they managed to maintain pluripotency better in space. In our present study, with time‐lapse imaging, we showed that space µg promoted the regeneration capacity and more dynamic behaviour of iPSCs. We speculated that the spreading of cells may prevent their overgrowth and differentiation. This could be one mechanism employed by iPSCs to better maintain their pluripotency under μg condition. Because the TZ‐1 cargo spacecraft did not travel back to earth, so we were not able to further analyse their physiological property of iPSCs survived the space µg in details. Further experiments in real µg should be carried out to confirm this phenomenon and reveal the underlying mechanism.

## CONFLICT OF INTEREST

The authors declare that they have no conflict of interest.
