# Supplementary figures and images for: Real microgravity condition promoted regeneration capacity of induced pluripotent stem cells during the TZ‐1 space mission
Source: Cell Prolif. 2019 Feb 6;52(3):e12574. doi: 10.1111/cpr.12574 (PMC6536455; doi:10.1111/cpr.12574)

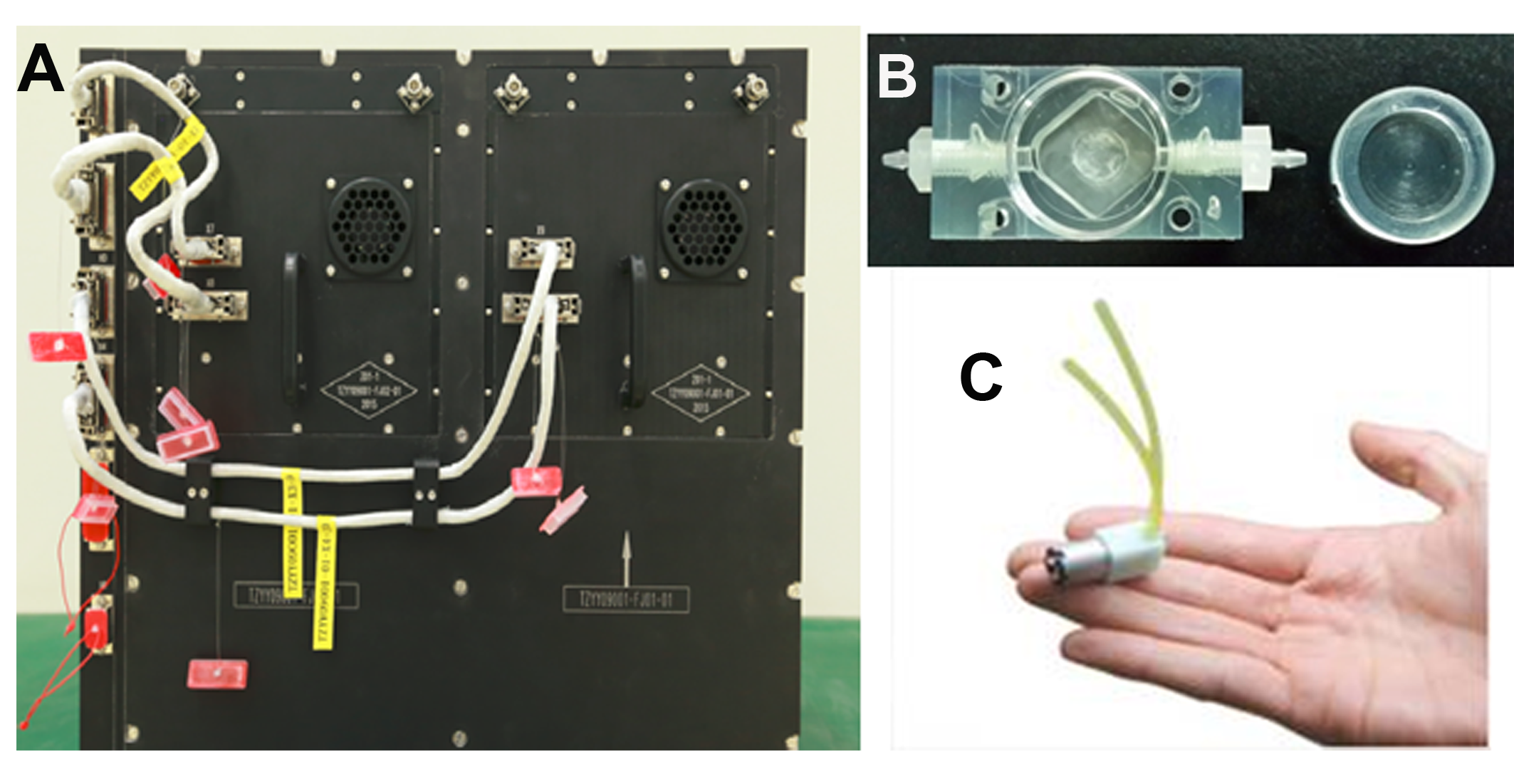

Supplement: Supplementary file 1 [file CPR-52-e12574-s001.tif]

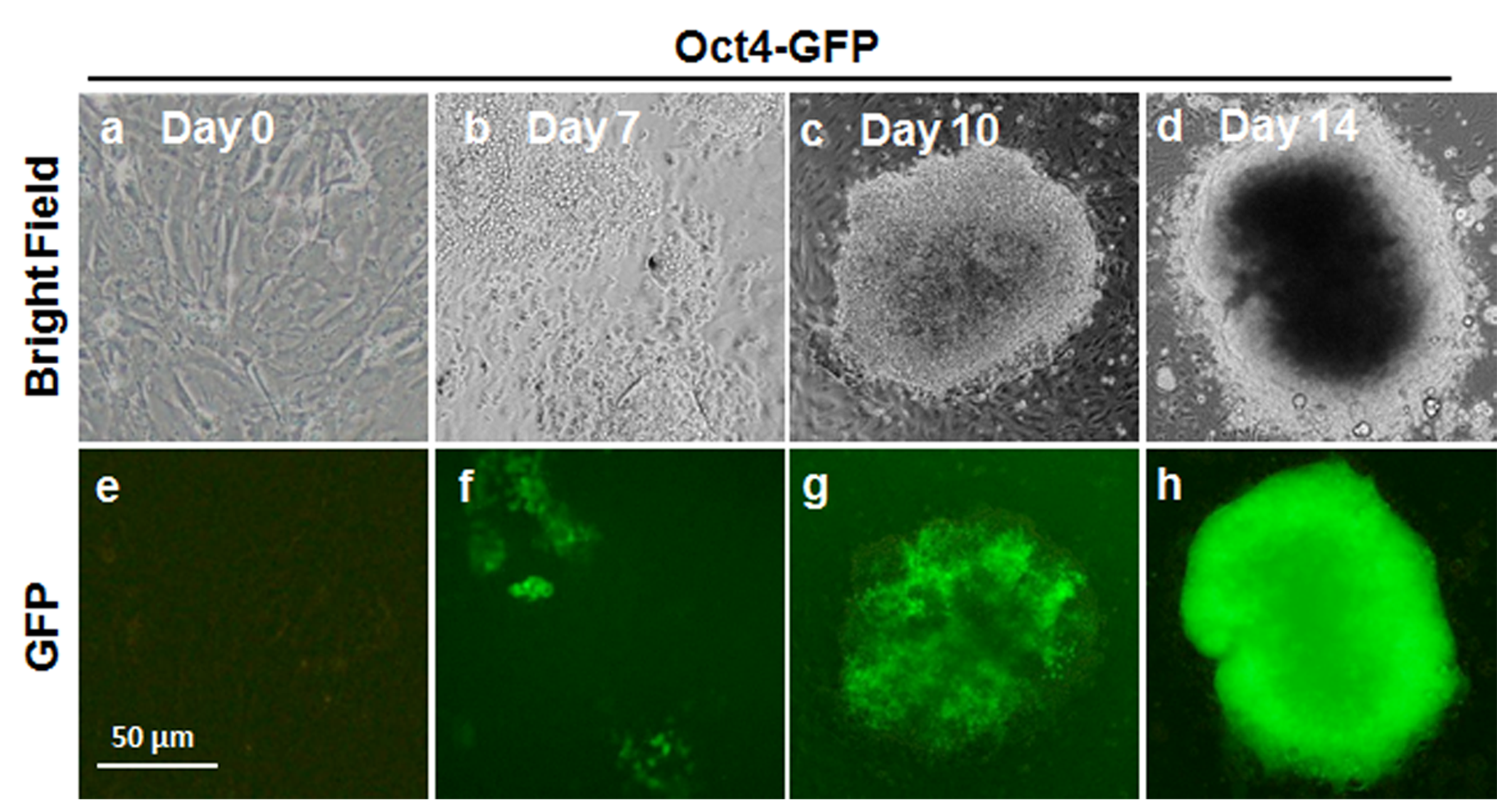

Supplement: Supplementary file 2 [file CPR-52-e12574-s002.tif]

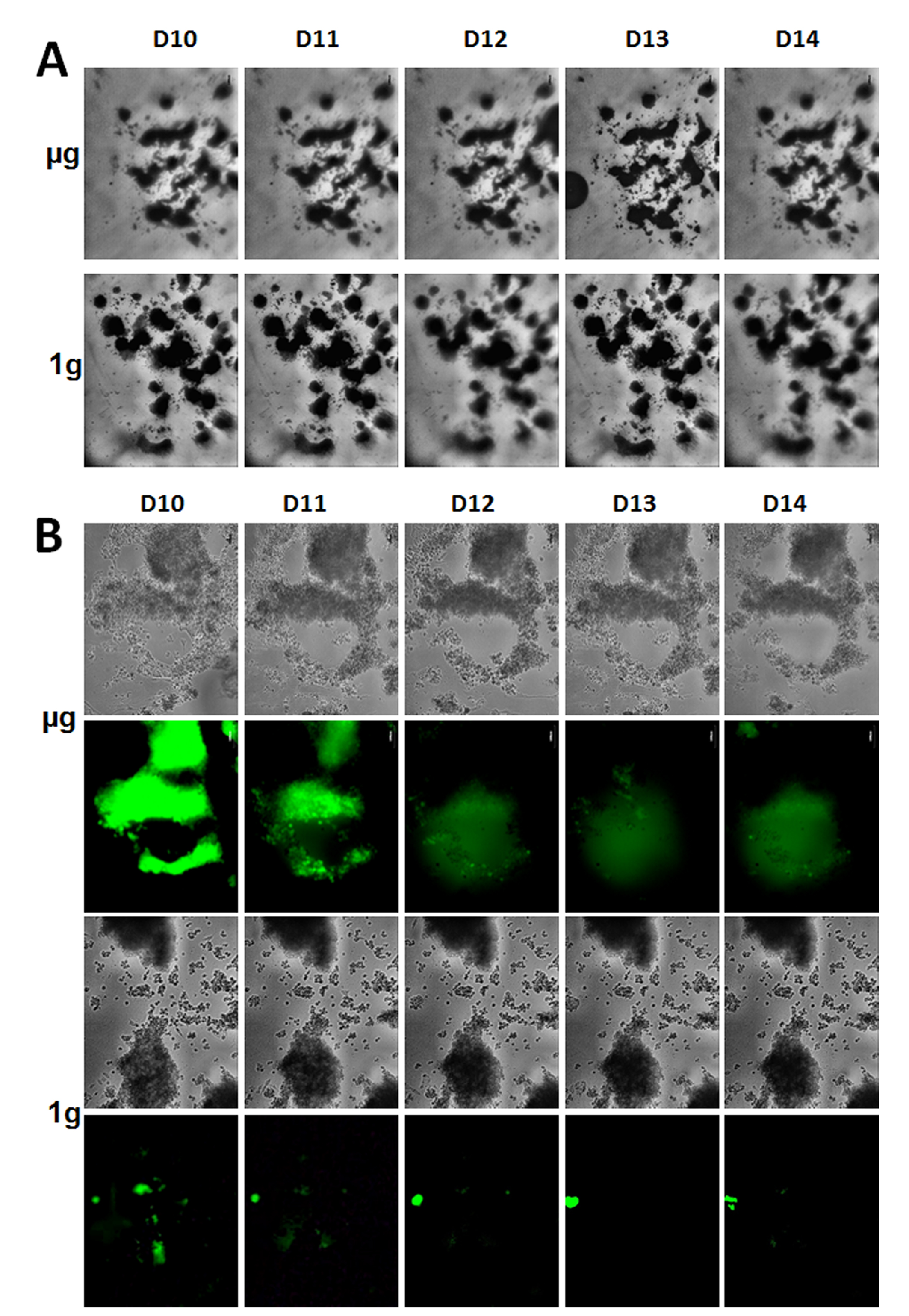

Supplement: Supplementary file 3 [file CPR-52-e12574-s003.tif]
