# Supplementary material for: Real microgravity condition promoted regeneration capacity of induced pluripotent stem cells during the TZ‐1 space mission
Source: Cell Prolif. 2019 Feb 6;52(3):e12574. doi: 10.1111/cpr.12574 (PMC6536455; doi:10.1111/cpr.12574)
Supplement: Supplementary file 4 [file CPR-52-e12574-s004.docx]

**Supplementary information**

## Real microgravity condition promoted dynamic migratory behaviors of induced pluripotent stem cells during the TZ-1 space mission

Running title: Time-lapse imaging study of iPSCs in real µg

*^,1^Jin Zhou, *^,1^Xiao-Hui Dong, *^,1^Feng-Zhi Zhang, ^1^Hui-Min Zhu, ^1^Tong Hao, ^1^Xiao-Xia Jiang, ^2^Wei-Bo Zheng, ^2^Tao Zhang, ^3^Pei-Zhe Wang, ^#,1^Hong Li, ^#,3^Jie Na, ^#,1^Chang-Yong Wang

**Supplementary Methods**

**Murine iPSCs generation and culture**

For mouse iPSCs generation, mouse tail fibroblasts or embryonic fibroblasts (MEF) were transfected with PB-CAG-mOCKS plasmid [1] (a generous gift from Sanger Institute, Cambridge, UK). Oct4-GFP iPSCs were reprogramed from fibroblasts derived from OG2 transgenic mouse carrying Oct4 promoter and enhancer driving GFP expression [2]. Mouse Oct4-GFP iPSCs were routinely cultured on inactivated MEF feeders with iPSC medium, which contained DMEM high glucose basal medium supplemented with 20% FBS, 4 mM L-glutamine, 1× non-essential amino acids (NEAA), 1×Glutamax, 1% antibiotic solution (penicillin/streptomycin), 0.1 mM β-mercaptoethanol, and 10 ng/mL LIF (Sinobiological). The medium was changed daily and cells were passaged 1:5 every 3 days using 0.05% trypsin/EDTA. The iPSCs condition medium (1 days) was gathered during the cell culture process and stored at 4 ℃ for the following iPSCs loading experiment.

**Preparation of the culture units of spaceflight bioreactor**

The cell culture units, which pre-treated with Matrigel (15 ug/ml) for 2 hours at 37 ℃. iPSCs clones at 10 hours after passaging were collected and transferred to the cell culture units of spaceflight bioreactor 28 hours before launch. The cell culture units were slowly infused with medium and discharged the bubbles. The medium bag was infused with the mixture of 20 ml iPSCs conditioned medium and 20 ml fresh medium. The medium bag was balanced in the 5% CO_2_ incubator overnight. The cell culture units connected with their corresponding medium bag and empty waste liquid bags were fixed to the bioreactor of TZ-1 spacecraft 12 hours before launch. The iPSCs medium was changed every day during the following experiment process after launch of TZ-1.

**Space** **Bioreactor System**

The Space Bioreactor System in this study were self-designed and developed by Shanghai Technological Physics Institute of Chinese Academy of Sciences. It can reach the acquirements of spatial dynamic cultivation of biological samples. More importantly, it firstly realizes the on-orbit microscopic observation of the samples continuously in the whole experimental process.

The Space Bioreactor System included 48 cell culture units. The installed cameras of white-light microscope and fluorescence microscope picked photos of 3 different fields of cells in the cell units automatically or according the uploaded control commands. Data were down-transferred to control center of TZ-1 mission every day.

**TZ-1 mission**

TZ-1 spacecraft launched on 19:41 April 20^th^ 2017, flying about 380 kilometers above the earth for 5 months in space. After completion of the space laboratory tasks, TZ-1 was controlled to deorbit and burn into the space atmosphere at 22^th^ September.

**Statistics:**

The size of iPSCs clone in the images was calculated by each clone area with Image J 2.0 software. Results were presented as the mean±SD. Data were analyzed using Graph Pad Prism 5. Statistical differences were evaluated by Student’s t-test analysis. Values were set as significant where *p*< 0.05.

**Acknowledgments:**

The biology experiments in the TZ-1 mission were organized by the Technology and Engineering Center for Space Utilization (CSU), Chinese Academy of Sciences. This work was supported by the Chinese Manned Space Flight Technology Project (TZ-1). This work was also supported by Natural Science Foundation of China (NSFC) grant 31320103914, U1601221 and National Key R&D Program of China grant 2016YFC1101303 to CW, the National Basic Research Program of China grant 2016YFE0204400, and NSFC grant 81571619 to HL, the National Key R&D Program of China grant 2017YFA0102802, NSFC grant 91740115, 31771108 to JN, the National Key Research and Development Program of China grant 2017YFA0106100 and NSFC for the Excellent Youth Scholars grant 81622027 to JZ.

**References:**

1. Wang, W., et al., *Rapid and efficient reprogramming of somatic cells to induced pluripotent stem cells by retinoic acid receptor gamma and liver receptor homolog 1.* Proc Natl Acad Sci U S A, 2011. **108**(45): p. 18283-8.

2. Szabo, P.E., et al., *Allele-specific expression of imprinted genes in mouse migratory primordial germ cells.* Mech Dev, 2002. **115**(1-2): p. 157-60.

**Supplementary Figure legends:**

**Supplementary Figure 1. The Bioreactor in the TZ-1 mission.**

A. The appearance of the Bioreactor. The culture unit (B) and micro-peristaltic pumps (C) in (A).

**Supplementary Figure 2. Bright field and GFP channel images showing the process of Oct4-GFP iPSC generation.**

A-D. Bright field images of MEF reprogramed to iPSC clones. E-H. GFP channel images of MEF reprogramed to iPSC clones. Note that in E, Oct4-GFP fibroblasts do not show any GFP fluorescence, while in H, Oct4-GFP iPSC clone has strong GFP fluorescence.

**Supplementary figure 3. The images of Oct4-GFP iPSCs in the ug and 1g groups at D10-14.**

A. The time-lapse images of iPSCs in μg and 1g at D10-14 of experiment (5X magnification).

B. Bright field and GFP channel images of iPSCs in μg and 1g at D10-14 of experiment (20X magnification).
